# Anti-Inflammatory and Wound Healing Properties of Different Honey Varieties from Romania and Correlations to Their Composition

**DOI:** 10.3390/life14091187

**Published:** 2024-09-20

**Authors:** Andreea Iosageanu, Laura Mihaela Stefan, Oana Craciunescu, Anisoara Cimpean

**Affiliations:** 1 Faculty of Biology, University of Bucharest, 91-95, Splaiul Independentei, 050095 Bucharest, Romania; andreea.iosageanu@incdsb.ro (A.I.); anisoara.cimpean@bio.unibuc.ro (A.C.); 2 National Institute of Research and Development for Biological Sciences, 060031 Bucharest, Romania; laura.stefan@incdsb.ro

**Keywords:** honey, wound healing, proliferation, anti-inflammatory activity, cytokines, epithelialization

## Abstract

The complex composition of honey plays a crucial role in wound healing, exhibiting varying effects at different stages of the healing process. This study investigated seven honey varieties sourced from different regions of Romania using in vitro experimental models developed in macrophage-like, fibroblast, and keratinocyte cell lines to explore the mechanisms by which honey promoted the healing process. This study assessed the impact of honey on inflammatory cytokine production in macrophage-like cells, cell proliferation and collagen synthesis in fibroblasts, and cell proliferation and migration in keratinocytes. Additionally, correlation analysis was conducted to examine the relationship between honey composition and its biological properties. Honey varieties presented both anti- and pro-inflammatory effects. Moreover, they displayed dose-dependent pro-proliferative effects, stimulating collagen synthesis and cell migration, thereby enhancing the re-epithelialization process. The Pearson coefficient analysis indicated a strong positive correlation between biological activities and phenolic content. Additionally, there was a medium positive correlation with the ascorbic acid content and a medium negative correlation with the glucose content in the different honey varieties. Romanian honey varieties rich in phenolics showed potential in modulating inflammation, proliferation, collagen synthesis, and cell migration, suggesting their suitability for further evaluation and development of innovative dressings for skin tissue regeneration.

## 1. Introduction

The alteration in skin integrity can compromise the skin barrier function, leading to increased permeability and susceptibility to pathogens. In severe cases, such disruptions can result in systemic complications, potentially endangering the life of the patient [1].

Healing of a wound is a complex biological process involving a variety of cell types, such as fibroblasts and keratinocytes, which secrete cytokines and extracellular matrix (ECM) components while also undergoing proliferation, migration, and differentiation to restore the damaged tissue to its pre-injury state [2]. Mouse models have long been used to understand the mechanisms of human pathologies as the overall structure of the immune system in mice is quite similar to that in humans, and almost all mice genes share functions with human genes [3].

Honey emerged as a promising candidate for wound care and has been utilized in the treatment of wounds across various cultures since ancient times [4]. It has a complex composition, rich in simple sugars, mainly fructose (~40%) and glucose (~30%), but it also contains low quantities of a plethora of bioactive compounds, such as phenolic acids, flavonoids, carotenoids, vitamins, organic acids, amino acids, enzymes, and minerals [5]. Previous in vitro and in vivo studies revealed that its medicinal properties, such as its antioxidant [6], antimicrobial [7], anti-inflammatory [8], and antitumor [9] potential, could be influenced by the content of certain constituents.

Clinical trials demonstrated that honey lowered the glycemic index in type 2 diabetes patients [10] and the duration of diarrhea in children with gastroenteritis [11] and also reduced the inflammatory response during wound healing [12] and served to develop honey-based platforms for controlled release with a beneficial effect on chronic wounds [13].

Honey-based products for wound healing are commercially available, with MEDIHONEY^®^ being a notable example. This Manuka honey-based formulation was the first biologically derived product approved by the Federal Drug Administration (FDA) for wound management [14], and other commercial honey-based dressings are under development.

Scientific studies indicated that honey was suitable for the treatment of a wide range of wounds, from burns and surgical incisions to open wounds, and it created a protective barrier at the healing site due to its viscosity and osmolarity [15]. It has been proven that honey polysaccharides can effectively regulate moisture due to their high swelling capacity, and they can attract lymph fluid to the wound bed, facilitating proteases contribution to debridement along with nutrition and epithelialization improvement [16]. In addition, honey-impregnated dressings exerted powerful antimicrobial activity and reduced antibiotic administration and hospitalization in diabetic foot patients [17]. The possible mechanism of preventing the accumulation of microorganisms was related to the synergistic action of sugars, gluconic acid responsible for a low pH between 3.2 and 5.5, and hydrogen peroxide, as well as the antimicrobial peptide bee defensin-1 [18].

Honey also exerted antioxidant activity to varying extents, which was mainly dependent on its phenolic content due to its known free radical scavenging capacity and modulation of intracellular reactive oxygen species (ROS) production through hydrogen atom transfer and single-electron transfer mechanisms [6]. Similarly, honey polyphenols could positively or negatively modulate the expression and secretion of pro-inflammatory cytokines at the wound site [19], which play a beneficial role in acute wound healing and are able to eliminate foreign debris and dead cells [20]. In turn, in chronic wound healing, the downregulation of pro-inflammatory cytokines, such as tumor necrosis factor α (TNF-α), interleukin 8 (IL-8), and interleukin 6 (IL-6), at both gene and protein expression is a major requirement during the therapeutic strategy to prevent the cascade of inflammatory mediator production at the wound sites [21].

The activation of fibroblast proliferation by growth factor release and new ECM synthesis together with skin ECM protection against protease action were favored by the acidic environment provided by honey [22]. It was also reported that honey promoted keratinocyte migration through the mediation of hydrogen peroxide production and the increase in intracellular calcium ion concentration for a rapid wound closure [23]. Still, the mechanisms are not fully understood.

Taking into account all these studies, the hypothesis of this study was that honey varieties have different properties according to their composition, namely, phenolic content, and as a result, honey might be more beneficial in certain phases of the complex wound healing process or wound type. Romanian honey varieties were scarcely investigated for their biological effect and utility in skin wound healing. To the best of our knowledge, this is the first study aiming to investigate the effect of seven Romanian honey varieties on the main phases of the wound healing process using in vitro experimental models of inflammation, proliferation, and remodeling developed in macrophage-like cells and skin cells (fibroblasts, keratinocytes) and to correlate their beneficial role to chemical composition.

## 2. Materials and Methods

### 2.1. Honey Sampling

**Figure 1:**
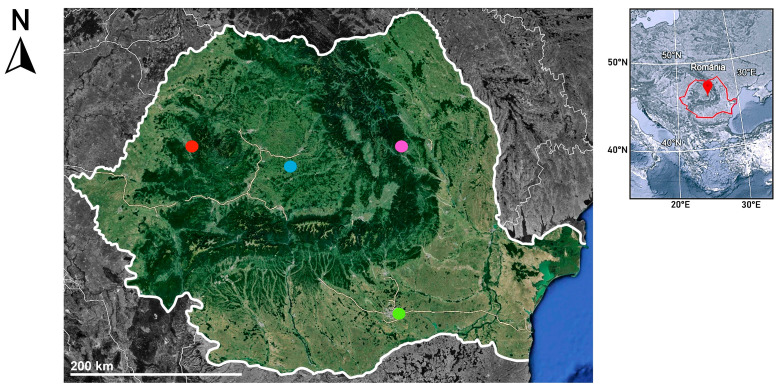
Locations of collected honey samples: green—linden honey (LH) and honeydew honey (HD), blue—sunflower honey (SH) and plain multifloral honey (MH1), pink—mountain multifloral honey (MH2), red—mountain multifloral honey (MH3) and meadow multifloral honey (MH4) (maps created using Google Earth, accessed on 25 July 2024).

Honey varieties of linden (LH), sunflower (SH), plain multifloral (MH1), mountain multifloral (MH2 and MH3), meadow multifloral (MH4), and honeydew (HD) were obtained directly from beekeepers from different regions of Romania (Figure 1) and stored at 4 °C.

The samples were chemically characterized, presenting 105.15–288.02 µg gallic acid equiv. (GAE)/g of total phenolic content, 0.65–6.26 µg quercetin equiv. (QE)/g total flavonoid content, 2.20–43.76 µg/g ascorbic acid, 279.49–329.31 mg/g glucose, and 342.42–418.11 mg/g fructose content [7] (Supplementary Table S1).

For cell culture experiments, honey samples were diluted in ultrapure water by stirring at 40 °C for 10 min, and then they were sterile filtered using 0.22 µm membranes. The dry mass of each honey sample was determined following dilution in ultrapure water and filtration.

### 2.2. Cell Lines and Cell Culture Conditions

Mouse NCTC clone L929 fibroblasts (ECACC, Sigma-Aldrich, Taufkirchen, Germany) were grown in Minimal Essential Medium (MEM) supplemented with 10% fetal bovine serum (FBS) and a 1% Penicillin–Streptomycin–Neomycin mixture (PSN) in T25 flasks under standard conditions in a humid atmosphere of 5% CO_2_/95% air at 37 °C. Human HaCaT keratinocytes (AddexBio Technologies, San Diego, CA, USA) and human THP-1 monocytic leukemia cells (ATCCs, Manassas, VA, USA) were grown in Roswell Park Memorial Institute (RPMI) 1640 medium supplemented with 10% FBS and 1% PSN in T75 flasks under standard conditions in a humid atmosphere of 5% CO_2_/95% air at 37 °C. All cell culture supplies were procured from Sigma-Aldrich (Germany) unless stated otherwise.

### 2.3. In Vitro Experimental Model of Inflammation

#### 2.3.1. Cell Treatment

Human THP-1 monocytes were seeded in 24-well plates at a cell density of 5 × 10^5^ cells/well and differentiated with 100 ng/mL phorbol 12-myristate 13-acetate (PMA) for 72 h to acquire the macrophage-like phenotype [24]. The culture medium was then replaced with fresh medium containing varying concentrations of honey samples (0.1–5 mg/mL) and incubated overnight. Cell viability was assessed using the Neutral Red assay as described below to determine honey biocompatibility. Following viability tests in THP-1-derived macrophage, L929 fibroblast, and HaCaT keratinocyte cultures, it was determined that the optimal honey concentration for inflammation, migration, and collagen synthesis tests was 0.5 mg/mL.

In the inflammation model, THP-1 monocytes were seeded and treated with PMA as previously described, followed by incubation with 10 ng/mL lipopolysaccharide (LPS) to induce inflammation and 0.5 mg/mL honey samples. After 24 h, the cell growth medium was harvested and centrifuged at 1000× *g* for 10 min, and then the supernatants were stored at −20 °C for cytokine level analysis, while the cell monolayer was subjected to a cell viability test. Untreated cells served as the negative control, while THP-1-derived macrophages exposed to LPS were considered the positive control.

#### 2.3.2. Cell Viability by the Neutral Red Assay

At the end of the incubation period, cell viability was evaluated by the Neutral Red assay [25]. Briefly, the culture medium was removed, and 100 μL Neutral Red stock solution diluted in culture medium at a final concentration of 0.05 mg/mL was added to the cell monolayer followed by 3 h incubation. Then, the cells were washed with 0.1 M phosphate-buffered saline (PBS), pH 7.4, and incubated with a fixative solution for 3 min. A solubilization solution of ethanol/deionized water/glacial acetic acid in a ratio of 50:49:1 (*v*/*v*/*v*) was added, and the plate was incubated and gently shaken at room temperature for 15 min. The absorbance of the colored solution was measured at a wavelength of 540 nm using a Spectrostar Nano microplate reader (BMG Labtech, Ortenberg, Germany).

#### 2.3.3. Quantification of Pro-Inflammatory Cytokine Production by ELISA

The level of pro-inflammatory cytokines, TNF-α and IL-8, was measured in supernatants using a specific sandwich ELISA kit, according to the manufacturer’s instructions (R&D Systems Inc., Minneapolis, MN, USA). The absorbance of the colored solution was read at 450 nm using a Spectrostar Nano microplate reader (BMG Labtech, Ortenberg, Germany). The results were normalized to cell viability values.

### 2.4. In Vitro Experimental Model of Skin Cell Proliferation

#### 2.4.1. Cell Treatment

Mouse L929 fibroblasts and human HaCaT keratinocytes were seeded at a density of 4 × 10^3^ and 6 × 10^3^ cells/well, respectively, in 96-well plates, and incubated in standard conditions overnight. Then, the growth medium was replaced with fresh medium supplemented with different concentrations of honey samples (0.1–50 mg/mL), and the plates were incubated for 24 and 48 h. Untreated cells served as the negative control, while the cells treated with 0.0001% H_2_O_2_ served as the positive control.

#### 2.4.2. Cell Viability and Proliferation by the Neutral Red Assay

Cell viability and proliferation were evaluated by measuring the increase in absorbance observed at the 24 and 48 h time points using the Neutral Red assay, as described above. It is postulated that the absorbance values correlate with the number of viable cells [26].

#### 2.4.3. Cell Morphology by Giemsa Stain

To assess cell morphology, L929 and HaCaT cells were seeded at a density of 2 × 10^4^ and 3 × 10^4^ cells/well, respectively, in 24-well plates, and incubated in standard conditions overnight. Honey samples were added in different concentrations, as presented for cell proliferation, and the plates were incubated for 48 h. Then, cells were fixed in methanol and stained with Giemsa. Images were acquired with an Axio Star microscope (Carl Zeiss, Oberkochen, Germany).

### 2.5. In Vitro Experimental Model of Wound Healing

The capacity of honey to stimulate cell migration and in vitro wound healing was evaluated using a scratch assay. HaCaT keratinocytes were seeded at a cell density of 1 × 10^5^ cells/well in 24-well plates and grown until they reached subconfluence. A scratch was created using a 200 μL sterile pipette tip, and cells were washed with PBS. Then, cells were treated with honey samples diluted in fresh medium supplemented with FBS at a concentration of 0.5 mg/mL. Untreated cells served as a negative control. Images were acquired before adding the samples (t = 0 h) and after 24 h of cultivation in standard conditions (t = 24 h). The percentage of the wound gap was determined for the control and study groups at each incubation time using ImageJ 1.53a software.

### 2.6. In Vitro Experimental Model of Collagen Synthesis

#### 2.6.1. Cell Treatment

L929 fibroblasts were seeded at a density of 5 × 10^4^ cells/well in 24-well plates and incubated in standard conditions for 24 h. Then, cells were treated with honey samples at a concentration of 0.5 mg/mL for 72 h. The culture medium was harvested and centrifuged at 1000× *g* for 10 min, and the supernatants were stored at −20 °C for collagen secretion analysis, while the cell monolayer served to assess cell viability. Untreated cells served as the negative control.

#### 2.6.2. Quantification of Type I Collagen Secretion by ELISA

The level of collagen synthesis was measured in supernatants using a specific competitive ELISA kit for type I collagen, according to the manufacturer’s instructions (MyBioSource, San Diego, CA, USA). The absorbance of the colored solution was read at 450 nm using a Spectrostar Nano microplate reader (BMG Labtech, Ortenberg, Germany). The results were normalized to cell viability values.

### 2.7. Statistical Analysis

All the experiments were performed in triplicate. The results are represented as mean ± standard deviation (SD) (*n* = 3). Statistical significance between control–sample and sample–sample pairs of interest was calculated using a two-tailed two-sample Student’s *t*-test (Microsoft Excel 2019), assuming that the populations have equal variances. The differences were considered statistically significant at *p* ≤ 0.05. The relationship between variables was analyzed by calculating the Pearson correlation coefficient.

## 3. Results

### 3.1. The Effect of Honey on the Inflammatory Phase of Wound Healing

**Figure 2:**
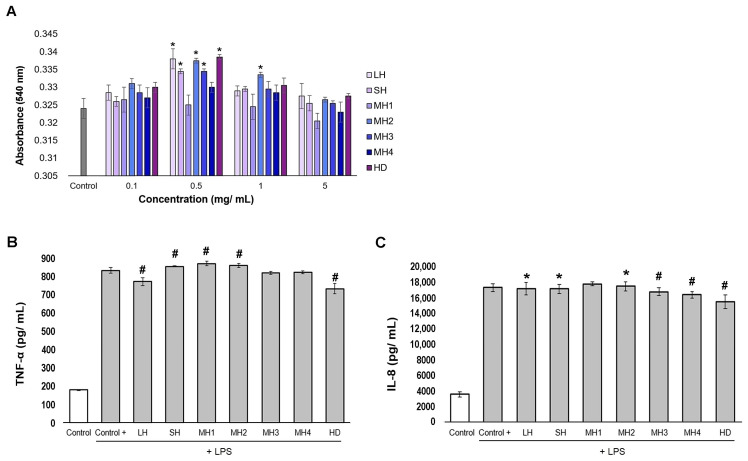
Cell viability of LPS-stimulated THP-1-derived macrophages treated with different concentrations of honey varieties for 24 h, as determined by the Neutral red assay (**A**). Secretion of TNF-α (**B**) and IL-8 (**C**) after 0.5 mg/mL honey treatment for 24 h, as determined by ELISA. Untreated cells served as negative control. LPS-treated cells served as positive control. Data represent the mean ± SD (*n* = 3). * *p* < 0.05; # *p* < 0.01 compared to positive control.

An in vitro experimental model in human THP-1-derived macrophages was designed to evaluate the effect of honey samples on the inflammatory milieu. Data showed that all samples were cytocompatible (cell viability values > 80%) in the range of tested concentrations of 0.1–5 mg/mL. Significantly (*p* < 0.05) higher values of absorbance ranging between 0.330 and 0.338 were registered for a concentration of 0.5 mg/mL honey compared to that of the untreated control (0.325). The results are presented in Figure 2A.

Data showed that HD honey and LH varieties exhibited significant (*p* < 0.01) inhibitory effects on TNF-α production and cells secreting 733.15 pg/mL and 771.49 pg/mL TNF-α, respectively, compared to the LPS-treated cells (832.68 pg/mL) (positive control) (Figure 2B). In turn, SH, MH1, and MH2 slightly stimulated TNF-α secretion (854.21–870.81 pg/mL).

The production of IL-8 was significantly (*p* < 0.05) inhibited by HD, recording a value of 15,419.16 pg/mL, followed by MH4 with 16,449.33 pg/mL, MH3 with 16,811.00 pg/mL, and SH and LH samples with 17,177.80 pg/mL and 17,195.23 pg/mL, respectively, compared to the LPS-treated cells (17,342.62 pg/mL) (Figure 2C). The sole sample that stimulated IL-8 secretion was MH2 up to a value of 17576.15 pg/mL.

All these results showed that HD honey could lower the production of pro-inflammatory cytokines, TNF-α (12%) and IL-8 (11%), and LH had a similar effect on TNF-α (7%), while MH3 and MH4 diminished IL-8 production (3–5%) in inflamed human macrophage-like cells. A stimulatory effect on TNF-α secretion was found for SH, MH1, and MH2.

### 3.2. The Effect of Honey on the Proliferative Phase of Wound Healing

#### 3.2.1. Fibroblast Viability and Proliferation

**Figure 3:**
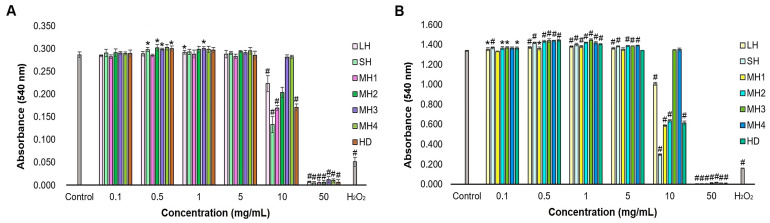
Cell viability and proliferation of L929 fibroblasts cultivated in the presence of different concentrations of honey samples for 24 (**A**) and 48 (**B**) h, as determined by the Neutral Red assay. Untreated cells served as negative control. Hydrogen peroxide-treated cells served as positive control. Data represent the mean ± SD (*n* = 3). * *p* < 0.05; # *p* < 0.01 compared to untreated control.

In this study, we developed an in vitro experimental model in mouse fibroblast-type cell culture treated with honey samples to evaluate the effect at the dermal level in terms of cell viability and proliferation. The results of L929 fibroblast viability in the presence of honey varieties are shown in Figure 3.

Data showed that the absorbance values, directly correlated to fibroblasts’ metabolic activity, increased in a dose-dependent manner, but to varying extents, depending on the honey sample. Thus, at 24 h of cultivation, LH, SH, MH1, MH2, and HD samples were not cytotoxic in a concentration range of 0.1–5 mg/mL, having similar or higher values compared to the untreated control. Also, MH3 and MH4 samples were not cytotoxic in a concentration range of 0.1–10 mg/mL compared to the untreated control. Higher concentrations of honey varieties induced a significant (*p* < 0.01) decrease in absorbance values compared to the untreated control, and the values were similar to the positive control of hydrogen peroxide at a concentration of 50 mg/mL honey.

Regarding cell proliferation, data showed that after 48 h of cultivation, the absorbance values were superior to those recorded after 24 h, indicating sustained stimulation of fibroblast proliferation in a time-dependent manner. Moreover, all honey varieties significantly (*p* < 0.01) stimulated cell proliferation in a concentration range of 0.5–5 mg/mL (1.341–1.449) compared to that of the untreated control (1.334), except for MH1 and HD samples. MH3 increased cell proliferation in the highest proportion at concentrations between 0.5 and 1 mg/mL. Higher concentrations significantly decreased fibroblast proliferation (*p* < 0.01), except for MH3 and MH4 varieties at a concentration of 10 mg/mL, which presented similar values to the untreated control.

**Figure 4:**
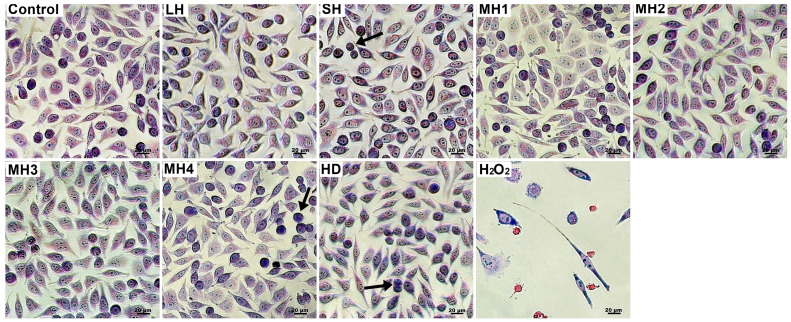
Cell morphology of L929 fibroblasts treated with 0.5 mg/mL honey varieties for 48 h. Normal phenotype and a few dividing round-shaped cells (arrows) were observed in honey-treated cells. Untreated cells served as negative control. Hydrogen peroxide-treated cells served as positive control. Giemsa staining. Bar scale = 20 µm.

Cell morphology observations were consistent with Neutral Red’s quantitative results. L929 fibroblasts from the untreated and honey-treated groups exhibited typical spindle-shaped morphology, with regular nuclei and nucleoli (Figure 4). The cells were well adhered, and a few dividing round-shaped cells were noticed. In the hydrogen peroxide-treated group, L929 cells revealed abnormal morphology with a larger appearance, losing their membrane integrity and severely reducing their adhesion capacity and cell density.

#### 3.2.2. Keratinocyte Viability and Proliferation

**Figure 5:**
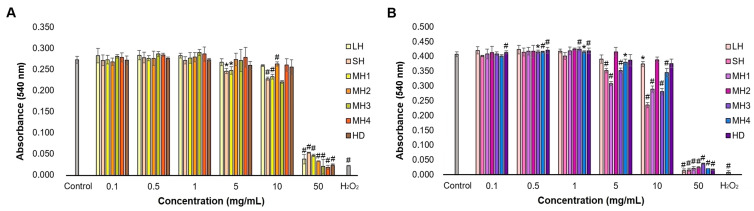
Cell viability and proliferation of HaCaT keratinocytes treated with different concentrations of honey samples for 24 (**A**) and 48 (**B**) h, as determined by the Neutral Red assay. Untreated cells served as negative control. Hydrogen peroxide-treated cells served as positive control. Data represent the mean ± SD (*n* = 3). * *p* < 0.05; # *p* < 0.01 compared to untreated control.

An in vitro experimental model developed in HaCaT keratinocytes treated with honey samples was used to evaluate their effect at the epidermal level, in particular on cell viability, proliferation, morphology, and migration. The results of cell viability and proliferation are presented in Figure 5.

At 24 h of cultivation, data showed that the absorbance values were similar to those of the untreated control cells for all honey varieties in a concentration range of 0.1–10 mg/mL, except for SH and MH1 at 5–10 mg/mL and MH2 at 10 mg/mL, indicating good interaction with human keratinocytes. Higher concentrations induced a significant (*p* < 0.01) decrease in absorbance values down to values recorded for the hydrogen peroxide-treated cells (positive control).

At 48 h of cultivation, MH3, MH4, and HD samples stimulated keratinocyte proliferation in a concentration range of 0.5–1 mg/mL (0.416–0.426) compared to the untreated control (0.404). Other honey varieties had no significant effect on cell proliferation and were similar to that of untreated control cells. Higher concentrations of honey ranging between 1 and 10 mg/mL significantly (*p* < 0.05) decreased keratinocyte proliferation at different extents, except for MH2- and HD-treated cells, which recorded a similar proliferation rate to that of the untreated cells. One could notice that stimulation of the keratinocyte proliferation rate, in the presence of honey varieties, took place at a smaller extent compared to that of fibroblast cells.

**Figure 6:**
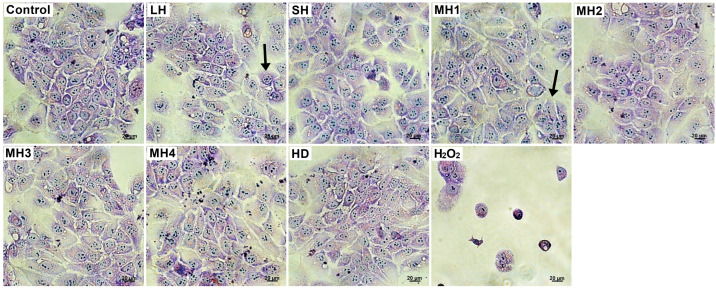
Cell morphology of HaCaT keratinocytes treated with 0.5 mg/mL honey varieties for 48 h. Normal phenotype and a few colonies (arrows) were observed in honey-treated cells. Untreated cells served as negative control. Hydrogen peroxide-treated cells served as positive control. Giemsa staining. Bar scale = 20 µm.

Cell morphology observations of honey-treated HaCaT keratinocytes showed the expression of a specific epithelial-like phenotype, with a typical polygonal shape and a tendency to form colonies (Figure 6). Good attachment to the culture plate and a cell density similar to that of the control cells appeared in all treated groups. In turn, hydrogen peroxide-treated keratinocytes exhibited cell shrinkage, round-shaped cells. and a severe decrease in cell density.

#### 3.2.3. Keratinocyte Migration

**Figure 7:**
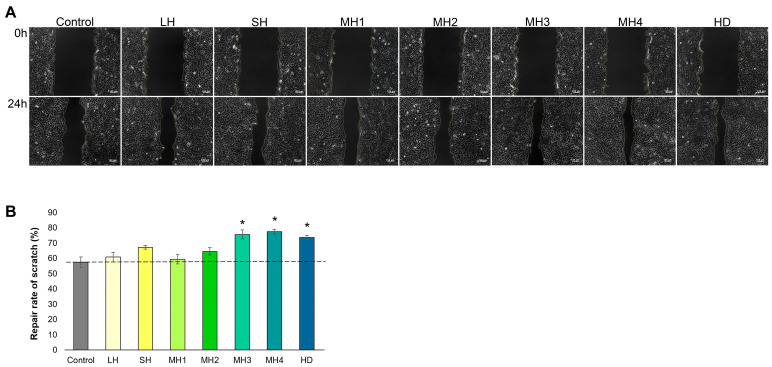
Phase contrast microscopy images of the scratch in HaCaT cells treated with 0.5 mg/mL honey varieties at t = 0 and t = 24 h ((**A**); scale bar = 100 µm). The repair rate of scratch was calculated using ImageJ 1.53a software (**B**). Untreated cells served as control. Data represent the mean ± SD (*n* = 3). * *p* < 0.05 compared to control cells.

The effect of honey treatment on keratinocyte migration was studied in a wound-healing experimental model by a scratch assay. The acquired images and calculated repair rates are presented in Figure 7.

Data showed that MH3-, MH4-, and HD-treated groups exerted a significantly (*p* < 0.05) higher repair rate of scratch, with values between 74 and 78%, compared to control cells (57%), indicating a high potential for improving wound closure. Also, LH, SH, MH1, and MH2 samples exerted a positive impact on the repair rate, but the values were not statistically significant (*p* > 0.05) in relation to control cells.

### 3.3. The Effect of Honey on the Remodeling Phase of Wound Healing

**Figure 8:**
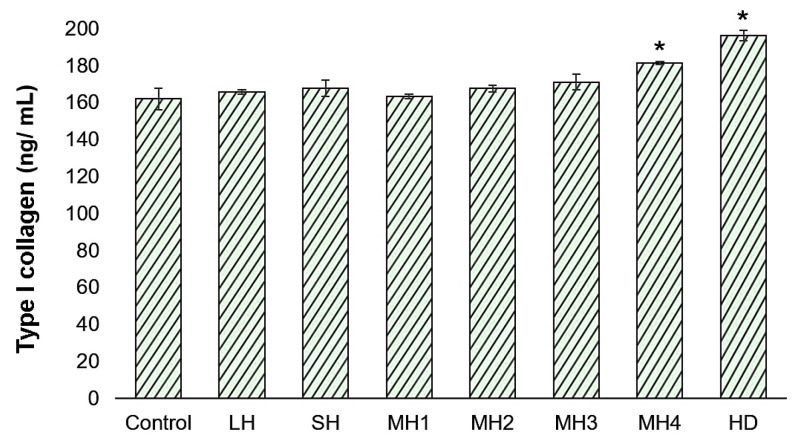
Levels of type I collagen synthesized by L929 fibroblasts treated with 0.5 mg/mL honey varieties for 72 h, as determined by ELISA. Data represent the mean ± SD (*n* = 3). * *p* < 0.05 compared to control cells.

Collagen type I synthesis is essential in the remodeling phase of wound healing [27]. The results obtained in the experimental model of L929 fibroblasts treated with honey varieties for 72 h are presented in Figure 8.

Data showed a significant increase (*p* < 0.05) of collagen synthesis in the cells cultivated in the presence of HD (196.47 ng/mL) and MH4 (181.59 ng/mL) compared to that in the untreated control cells (162.22 ng/mL). Other honey varieties presented similar values of collagen production to that of the untreated control with regard to collagen synthesis.

### 3.4. Analysis of the Correlation between Chemical Composition and Biological Properties

**Figure 9:**
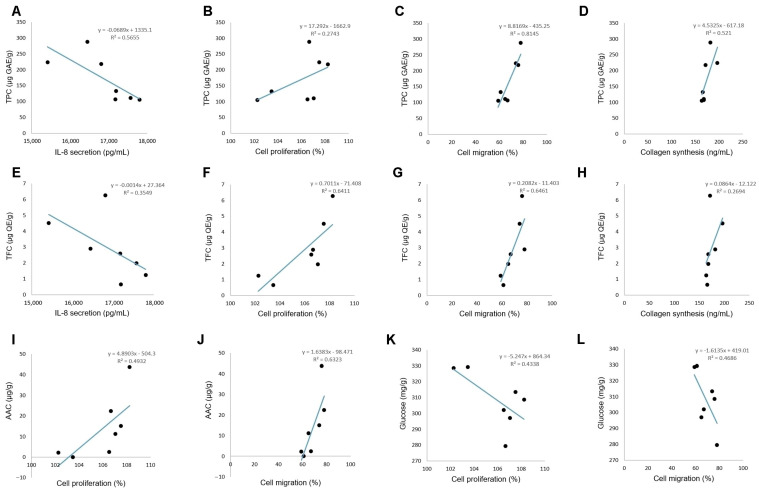
Scatter plot diagrams showing the correlation of total phenolic content (TPC) vs. IL-8 secretion (**A**), cell proliferation (**B**), cell migration (**C**), collagen synthesis (**D**); total flavonoid content (TFC) vs. IL-8 secretion (**E**), cell proliferation (**F**), cell migration (**G**), collagen synthesis (**H**); ascorbic acid content (AAC) vs. cell proliferation (**I**), cell migration (**J**); glucose content vs. cell proliferation (**K**), cell migration (**L**). Data represent the mean of three determinations (*n* = 3).

The total phenolic, flavonoid, ascorbic acid, fructose, and glucose content of honey varieties, on one side, and their biological activity, on the other side, were statistically analyzed to establish Pearson’s correlation coefficients (Table 1). The correlations between the main honey constituents and their biological activities are given in Figure 9.

Data showed that keratinocyte migration was strongly positively correlated with total phenolic (Figure 9C) and flavonoid content (Figure 9G), moderately correlated with ascorbic acid content (Figure 9J), and moderately negatively correlated with glucose content (Figure 9L). A strong correlation was also observed between fibroblast proliferation and flavonoid content (Figure 9F), while a medium positive correlation was found with total phenolic (Figure 9B) and ascorbic acid content (Figure 9I), and there was a medium negative correlation with glucose content (Figure 9K). Collagen synthesis was moderately positively correlated with total phenolic (Figure 9D) and flavonoid content (Figure 9H), while a poor correlation was found with ascorbic acid and glucose content. Furthermore, IL-8 secretion was moderately negatively correlated with total phenolic (Figure 9A) and flavonoid content (Figure 9E), while TNF-α secretion was poorly correlated with total phenolic, flavonoid, ascorbic acid, fructose, and glucose content.

## 4. Discussion

Skin wound healing is a complex process that occurs in four phases, hemostasis, inflammation, proliferation, and remodeling, which overlap and are regulated by molecules that trigger cellular events involved in cell proliferation, migration, and differentiation [28,29]. Once the injury has occurred, hemostasis begins in order to stop blood loss and prepare the wound bed for the next phases of healing [30]. Further, the blood vessels dilate and increase in permeability allowing inflammatory cells to enter the wound bed, which marks the start of the second phase of healing, inflammation [31].

The inflammatory phase requires the infiltration of neutrophils, macrophages, and lymphocytes to the wound bed to remove pathogens and cellular debris, as well as to release molecules that promote wound healing [32]. The molecules secreted by neutrophils, reaching first the wound bed, are mainly cytokines and growth factors that further recruit other immune cells [33] and activate local fibroblasts and epithelial cells [34]. TNF-α and pro-inflammatory interleukins IL-8 and IL-6 could act as activators of NF-kB and Toll-like 4 receptors involved in the initiation of inflammation via canonical signaling [35].

In the present study, the results obtained in an experimental model in vitro mimicking the inflammatory phase of wound healing confirmed that Romanian honey varieties possessed anti-inflammatory properties by decreasing TNF-α and IL-8 production up to 12% in human inflamed macrophage-like cells (Figure 2). According to the correlation analysis, this activity was mainly influenced by phenolic content and not by sugars (Table 1). Similar studies reported the inhibition of nitric oxide, as a mediator of inflammation in LPS- and interferon γ-inflamed murine RAW 264.7 macrophages by Malaysian honey phenolic extract, which is rich in ellagic and ferulic acid, myricetin, and hesperetin, demonstrating their role in anti-inflammatory activity [36]. However, LC-ESI-MS analysis indicated that some phenolics were present in conjugated forms, presenting sugar moieties, such as ellagic 3-O-glucoside. The study on the methanolic fraction of *Melipona beecheii* honey from Mexico, which is rich in polar phenolic acids, revealed that it was a potent anti-inflammatory agent compared to honey, emphasizing its role in relation to the mechanisms of protein denaturation and protease inhibition [37]. The main mechanism of action of honey phenolics was found in relation to their free radical scavenging capacity and lipid peroxidation chain inhibition, thus limiting the state of inflammation [38]. The modulation of ROS-triggered transcription factors, such as NF-kB, ERK1/2, and Nrf2, by honey flavonoids could control pro-inflammatory enzymes and cytokine gene expression involved in pathophysiological processes [19,39]. Still, no studies were found to correlate phenolic content to the anti-inflammatory potential.

Other in vitro studies showed that honey treatment led to the inhibition and/or stimulation of pro-inflammatory cytokines in a dose-dependent manner. Thus, 400 μg/mL of raw Manuka honey showed a 30% inhibition of TNF-α in rat neutrophils, while at 100 μg/mL, it stimulated TNF-α secretion [40]. It was also reported that Manuka honey stimulated the in vitro release of TNF-α after 10 mg/mL treatment of human monocytes isolated from peripheral blood [41]. Also, IL-8 secretion was efficiently inhibited by Taiwan forest honey in WiDr cells that harbored the R273H mutant of p53, while *Bidens pilosa* honey had a stimulatory effect [42]. In vivo studies revealed that honey pre-treatment of LPS-challenged mice could lower the levels of serum TNF-α [43]. The capacity of honey samples to inhibit or stimulate TNF-α and IL-8 secretion demonstrated their potential role in the modulation of Th1/Th2 balance via macrophage activation and the regulation of inflammatory and immune responses.

In the proliferative phase, fibroblasts undergo differentiation and secrete ECM components, playing a crucial role in wound contraction. This is succeeded by the proliferation, migration, and differentiation of epithelial cells, which collectively contribute to the restoration of epidermal integrity through a process known as epithelialization [44]. In the present study, all honey varieties demonstrated pro-proliferative properties stimulating up to 9% of the cell proliferation rate in fibroblast culture (Figure 3). This effect was correlated to their phenolic, flavonoid, and ascorbic acid content (Table 1), suggesting that the presence of these honey constituents might improve tissue regeneration. In turn, a medium negative correlation was observed between the glucose content of honey and fibroblast proliferation. The sugar content was responsible for creating an osmotic gradient and managing the wound exudate [45]. Other in vitro studies showed that the incubation of Tualang and gelam honey with human periodontal ligament fibroblasts and 3Y1 rat fibroblasts resulted in a 27 and 35% increase in cell proliferation, respectively [46]. Also, the treatment of 46 BR.1N human skin fibroblasts with Manuka, buckwheat, and acacia honey induced cell migration, helping the tissue repair activity [47]. However, high glucose concentration (50 mM) impaired the proliferation and migration of human gingival fibroblasts [48], while 30 mM glucose delayed human foreskin fibroblast migration, but not cell proliferation [49], and 20 mM glucose-induced oxidative stress, which slowed the replication of human endothelial cells [50].

At the end of the proliferative phase, keratinocytes undergo proliferation, migration, and differentiation, which help with the restoration of skin integrity in a process called re-epithelialization [51]. In the present study, MH4, MH3, and HD honey samples stimulated keratinocyte proliferation and greatly increased keratinocyte migration, positively influencing wound closure in vitro up to a rate of 78% at 0.5 mg/mL treatment (Figure 7). This finding was in line with the effect of a higher concentration of 200 mg/mL jujube honey treatment that almost repaired the scratch (99.21%) compared to untreated cells, showing 80% wound closure after 48 h of incubation [52].

Similar to fibroblast proliferation, the stimulation of keratinocyte migration was correlated to phenolic, flavonoid, and ascorbic acid content of honey varieties (Table 1). These compounds are known contributors to antioxidant activity, suggesting that the reduction in oxidative stress may represent a viable mechanism through which honey promotes wound healing. It was previously shown that following treatment of primary human dermal fibroblasts with Manuka honey, the phosphorylation of AMPK took place, and its activation determined the enhancement of mitochondrial function [53]. Additionally, elevated levels of Nrf2 protein were accompanied by the increased transcription of catalase and superoxide dismutase genes. It was hypothesized that AMPK activation promoted wound healing by restoring the energy balance during metabolic stress [54] and by enhancing cell migration through the modulation of the cytoskeleton [55]. Conversely, Nrf2 activation facilitated wound healing by reducing oxidative stress and promoting both cell proliferation and migration [56].

The final stage of wound healing, referred to as the remodeling phase, is a prolonged process that can extend over several years. This phase involves the substitution of type III collagen synthesized in the early stages with type I collagen, leading to a significant improvement in tissue strength and structural integrity [57,58]. Additionally, a reduction in metabolic activity [51] and a regression of newly formed blood vessels [59] was observed, and both processes contributed to the formation of mature ECM [27].

In the present study, HD and MH4 honey samples significantly stimulated type I collagen synthesis by up to 21% (Figure 8), correlating with high phenolic and flavonoid content (Table 1). A low correlation was found between collagen production and ascorbic acid content. Previously, the role of exogenous ascorbic acid in accelerating wound healing through antioxidant and anti-inflammatory activity [60] was highlighted, but its physiological concentration was found to be too low to ensure protective action against oxidation [61]. Therefore, supplementation with natural antioxidant compounds from honey could modulate the oxidative stress and TNF-α and IL-1β pro-inflammatory cytokine released from monocytes, which is involved in the regulation of certain matrix metalloproteinases (MMPs) expression, like MMP-1 and MMP-9, and collagen types I and IV degradation during wound healing [20,62,63]. A direct action of as low as a 0.02% concentration of stingless bee honey from Morocco was demonstrated to significantly downregulate MMP-1 expression in senescence fibroblast cells [64]. In addition, this study demonstrated that honey upregulated collagen mRNA expression in senescence cells, thus contributing to ECM structural integrity. These combined effects of honey are essential to achieve a balance between collagen metabolism suppression and collagen synthesis stimulation to promote a supportive environment for collagen accumulation and tissue repair.

Considering these mechanisms of action, other in vitro studies indicated that Turkey chestnut honey (*Castanea sativa*) has exhibited significant antioxidant and anti-inflammatory properties along with the ability to inhibit DNA damage in view of their high concentration of phenolic compounds [65,66]. New Zealand kanuka honey (*Kunzea ericoides*) exhibited immunostimulatory activity by enhancing the release of TNF-α from THP-1 cells [67]. South American ulmo honey (*Eucryphia cordifolia*) has also been associated with wound healing properties, which are attributed to its anti-inflammatory mechanisms [68] and substantial antioxidant and antimicrobial activity [69]. Malaysian gelam honey (*Melaleuca cajuputi*) enhanced corneal wound healing in experimental models [70]. These findings suggested that, despite the diverse botanical origins of honey, many varieties shared similar biological properties, including antioxidant, anti-inflammatory, and antibacterial effects, and were able to stimulate the late remodeling phase of wound healing.

**Figure 10:**
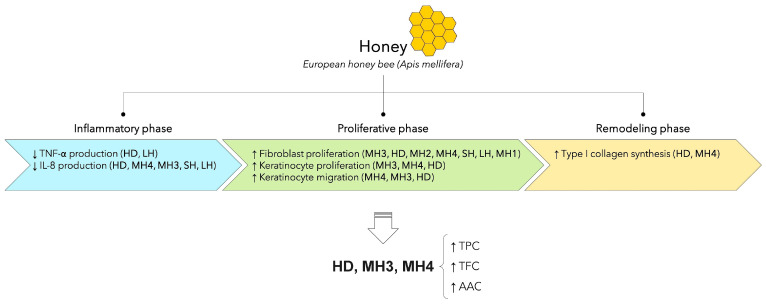
Scheme of Romanian honey variety properties in the main phases of wound healing and the selection of optimal samples in correlation with their composition.

In view of the results of the present study, a high correlation was established between total phenolics, flavonoids, and ascorbic acid content vs. fibroblast proliferation and keratinocyte migration, next to total phenolic vs. collagen synthesis (Table 1). A low or negative correlation was found between the phenolic content vs. pro-inflammatory cytokine secretion (Table 1). This indicated that phenolic compounds present in honey varieties might be important active agents for the main phases of wound healing, particularly in the late phases of proliferation and re-epithelialization or chronic wounds (Figure 10). Accordingly, HD honey and MH3 and MH4 samples with a high content of phenolics were selected as being optimal for wound healing. A similar report mentioned a positive role of honey in chronic wounds [71], while others showed a positive influence in all phases of wound healing [15].

## 5. Conclusions

In conclusion, HD honey and MH3 and MH4 varieties showed the best results in stimulating fibroblast proliferation, keratinocyte migration, and collagen synthesis in vitro. These activities were strongly or moderately positively correlated with their total phenolic content and moderately or weakly correlated with ascorbic acid content. Sugar content had a low correlation with these events. Also, the results indicated better effects in processes taking place in the re-epithelialization and remodeling phases of the wound healing process, thus providing a beneficial role mainly in tissue recovery or chronic wounds. Further studies are required on a broader range of varieties to facilitate the establishment of honey quality based on their phenolic compound content for therapeutic applications and their valorization in novel honey-based dressings for wound healing.

## Figures and Tables

**Table 1:** Pearson correlation coefficients between main honey constituents and the biological activities of the analyzed honey samples.

	TPC	TFC	Ascorbic Acid	Fructose	Glucose
TNF-α secretion	−0.465	−0.282	−0.110	0.181	−0.223
IL-8 secretion	−0.752	−0.596	−0.376	0.331	0.243
Fibroblast proliferation	0.524	0.801	0.702	−0.051	−0.659
Keratinocyte migration	0.903	0.804	0.795	−0.424	−0.685
Collagen synthesis	0.722	0.519	0.308	−0.034	−0.309

Green—strong correlation (>0.8), blue—medium correlation (0.5–0.8), white—poor correlation (<0.5); TPC—total phenolic content, TFC—total flavonoid content.

## Data Availability

The data presented in this study are available on request from the corresponding author. The data are not publicly available due to legal restrictions.

## Supplementary Materials

The following supporting information can be downloaded at: https://www.mdpi.com/article/10.3390/life14091187/s1, Table S1: The content of total phenolic (TPC), flavonoid (TFC), ascorbic acid, glucose and fructose content of various Romanian honey varieties.
