# Supplementary material for: Anti-Inflammatory and Wound Healing Properties of Different Honey Varieties from Romania and Correlations to Their Composition
Source: Life (Basel). 2024 Sep 20;14(9):1187. doi: 10.3390/life14091187 (PMC11432766; doi:10.3390/life14091187)
Supplement: Supplementary file 1 [file life-14-01187-s001.zip › life-3173667-supplementary.pdf]

Table S1. The total phenolic (TPC), flavonoid (TFC), ascorbic acid, glucose, and fructose content of various Romanian honey varieties.

**Sample**	**Geographical region**	**TPC[a] (µg GAE/g)**	**TFC[a] (µg QE/g)**	**Ascorbic acid[a] (µg/g)**	**Glucose[a] (mg/g)**	**Fructose[a] (mg/g)**
Linden honey (LH)	Muntenia	107.10 ± 2.59	0.65 ± 0.09	ND	329.31 ± 2.05	394.10 ± 2.56
Sunflower honey (SH)	Transylvania	132.52 ± 3.61	2.59 ± 0.52	2.47 ± 0.43*	302.07 ± 1.33	418.11 ± 1.28
Plain multifloral honey (MH1)	Transylvania	105.15 ± 0.39	1.24 ± 0.06	2.20 ± 0.10	328.62 ± 2.50	408.77 ± 1.96
Mountain multifloral honey (MH2)	Moldavia	110.63 ± 0.61	1.98 ± 0.23	11.20 ± 0.29	297.02 ± 2.93	398.85 ± 2.23
Mountain multifloral honey (MH3)	Crisana	218.02 ± 0.51	6.26 ± 0.49	43.76 ± 0.10	308.64 ± 1.78	418.02 ± 2.64
Meadow multifloral honey (MH4)	Crisana	288.02 ± 2.94	2.89 ± 0.83	22.42 ± 1.57	279.49 ± 1.30	342.42 ± 1.77
Honeydew honey (HD)	Muntenia	223.94 ± 2.45	4.52 ± 0.09	15.06 ± 0.30	313.4 ± 2.45	396.03 ± 1.15

[a]TPC determined by the Folin–Ciocalteu method, TFC determined by the $AlCl_3$ method, ascorbic acid determined by the 2,6 dichlorophenol-indo-phenol (DCPIP) method, and glucose and fructose content determined by HPLC [6].
